# Iron Lemonade

**Published:** 1887-11

**Authors:** 


### Iron Lemonade.

The tincture of iron injures the enamel of the teeth unless administered in great dilution. An exchange in publishing the following may not have thought of this, or else considered it such a palatable drink that the liability of injury to the teeth is of no comparable consequence:

"The following method of prescribing the muriated tincture of iron is recommended as being the most palatable, elegant, and altogether satisfactory ever yet offered to the public:

| | |
|---|---|
| Tincture of chloride of iron | 4 fl. drs. |
| Diluted phosphoric acid | 6 fl. drs. |
| Spirit of lemon | 2 fl. drs. |
| Syrup | 6 fl. ozs. |

Mix.

"Two teaspoonfuls in water after each meal. The spirit of lemon is preferred to the syrup of lemon, as the latter would render the mixture too acid. We have found the above, combined with pepsin, an excellent tonic when indications are associated with feeble digestive powers."

